# Semi-Empirical Approach to Evaluating Model Fit for Sea Clutter Returns: Focusing on Future Measurements in the Adriatic Sea

**DOI:** 10.3390/e26121069

**Published:** 2024-12-09

**Authors:** Bojan Vondra

**Affiliations:** Department of Communication and Space Technologies, Faculty of Electrical Engineering and Computing, University of Zagreb, 10000 Zagreb, Croatia; bojan.vondra@fer.hr

**Keywords:** Kullback–Leibler divergence, squared Hellinger distance, sea clutter, goodness of fit, exponentially distributed waiting times

## Abstract

A method for evaluating Kullback–Leibler (KL) divergence and Squared Hellinger (SH) distance between empirical data and a model distribution is proposed. This method exclusively utilises the empirical Cumulative Distribution Function (CDF) of the data and the CDF of the model, avoiding data processing such as histogram binning. The proposed method converges almost surely, with the proof based on the use of exponentially distributed waiting times. An example demonstrates convergence of the KL divergence and SH distance to their true values when utilising the Generalised Pareto (GP) distribution as empirical data and the K distribution as the model. Another example illustrates the goodness of fit of these (GP and K-distribution) models to real sea clutter data from the widely used Intelligent PIxel processing X-band (IPIX) measurements. The proposed method can be applied to assess the goodness of fit of various models (not limited to GP or K distribution) to clutter measurement data such as those from the Adriatic Sea. Distinctive features of this small and immature sea, like the presence of over 1300 islands that affect local wind and wave patterns, are likely to result in an amplitude distribution of sea clutter returns that differs from predictions of models designed for oceans or open seas. However, to the author’s knowledge, no data on this specific topic are currently available in the open literature, and such measurements have yet to be conducted.

## 1. Introduction

As discussed in [1], civilian coastline safety can be increased by monitoring gaps of inadequate coverage of the principle radars by employing lightweight Commercial Of The Shelf (COTS) radar sensors installed on mobile platforms, seeking for illegal vessels, presumably dim, manoeuvring and embedded in sea clutter. Due to the requirement for a small antenna profile, the operating frequency of these sensors lies in the X band and amplitude output is logarithmically rectified, resulting in the loss of phase information. A typical monitoring scenario involves tracking a highly manoeuvring Rigid Inflatable Boat (RIB), a dim target whose echo is significantly interfered with by sea clutter returns. During the process of algorithm performance evaluation, various tracking scenarios require the replication of clutter interference to a high degree of accuracy. Specifics of surveillance area, like the existence of islands and littoral environments, greatly affect local wind and wave characteristics. An example is the Croatian part of the Adriatic basin, which is a small and enclosed sea with more than 1300 islands, where surface wind waves are limited by fetch and wind duration, and depths cannot be neglected in most of the basin. For these reasons, the Adriatic Sea is an immature sea, with steeper waves than its counterpart in the ocean [2]. As, to the best of the author’s knowledge, measured clutter statistics of the Croatian part of the basin do not exist in the open literature, it is reasonable (to a certain extent) to expect clutter amplitude statistics that are different than those from models verified for the ocean, under the same wind conditions and particularly in littoral environments. Hence, in this specific context, replicating clutter interference necessitates modelling both the texture and speckle amplitude distributions, as well as the short- and long-term correlations. With this in mind, assessing the goodness of fit between the proposed models and the empirical data becomes crucial. However, through the rest of this paper, only clutter amplitude distribution is considered.

Goodness-of-fit measures are not always provided in the open literature in relation to sea clutter amplitude statistical fit. Some authors provide only visual goodness of fit, like in [3], where a proposed KK distribution is compared with a KA distribution in the tail region. The same dataset was used in [4] to extend the KK distribution with thermal noise, and a visual comparison with the K distribution was given. Other examples where only visual fit was presented are [5], where empirical amplitude distribution was fitted with the K distribution; ref. [6], where the authors fitted heavy-tailed empirical data with K, GP and Weibull distributions; ref. [7], where goodness of fit was given as the visual deviation of empirical data from the theoretical t distribution and an inverse Gaussian model in quantile–quantile plots; refs. [8,9], where the empirical amplitude distribution under the condition of short fetch was analysed and fitted with the K distribution; ref. [10], where empirical data were compared to Rayleigh, log-normal, K and Weibull distributions; and recently [11], where empirical data were modelled as a K distribution with noise, and the texture distribution was represented by gamma, inverse gamma, inverse Gaussian and log-normal distributions. Examples where a goodness-of-fit measure was provided by means of the chi-squared test are [12], where the fits of the K and Weibull distributions were tested, and [13], where Rayleigh and log-normal distributions were tested in addition to previously mentioned models. Other examples where goodness-of-fit measures were provided are [14], where an empirical cumulative distribution was used to perform Kolmogorov–Smirnov (KS) tests with the reference K distribution; ref. [15], where the goodness-of-fit measure for the proposed Compound Gaussian Inverse Gaussian (CGIG) texture model was given as the mean absolute quantile deviation; ref. [16], where the K-distribution fit was tested using a KS test and measure of fit was given as root mean square error; ref. [17], where the fit of Weibull, log-normal and Ricean Inverse Gaussian (RiIG) [18] distributions to the proposed CGIG distribution was measured using the Mean Square Error (MSE) criterion; and [19], where segmented sea clutter data in littoral environments were fitted with the K distribution and measure of fit was given as the absolute difference between the empirical and theoretical CDF. Recently, in [20], Bhattacharyya distance was used as a goodness-of-fit measure between empirical data and the K distribution with noise (including its variant with an extra Rayleigh component). The GP distribution with noise and the tri-modal discrete distribution were proposed in [21]. In [22], sea clutter data were fitted with Weibull, log-normal, generalised gamma, $G^{0}$ and α-stable distributions and comprehensive goodness-of-fit measures were provided, including KL divergence, Bhattacharyya distance and MSE, and finally, in [23], the authors fitted real sea clutter data with various distributions such as CGIG, GP and K distributions and goodness of fit was measured using KS distance and KL divergence, using both the empirical Probability Density Function (PDF) and the empirical CDF. Besides the papers referenced above, interested readers can find out more about K, KA, GP, Rayleigh, log-normal and Weibull distributions in [24,25,26,27,28], gamma and inverse gamma texture models in [29], $G^{0}$ distributions in [30] and α-stable distributions in [31].

It is interesting that, despite the extensive literature on clutter modelling, the author could not find any published goodness-of-fit tests that utilise the Wasserstein distance within the optimal transportation framework. However, ref. [32] provides a robust foundation for assessing goodness of fit between empirical data and a model in both univariate and multivariate cases, which involves solving the semi-discrete optimal transport problem. Additionally, ref. [33] establishes a Central Limit Theorem for semi-discrete distributions. These foundational contributions provide a strong basis for developing future goodness-of-fit tests for clutter modelling using the Wasserstein distance.

The contribution of this paper is the proposition of an estimator for KL divergence and SH distance that relies exclusively on the empirical and model CDFs, unlike the aforementioned methods that assess the goodness of fit using histogram binning of observed data. This work builds upon the methodology introduced in [34,35], where empirical CDFs of both distributions that were compared were employed in the empirical estimation of KL divergence and SH distance, respectively. Unlike empirical estimators, the estimator proposed in this work is semi-empirical and is restricted to univariate distributions. Although the empirical estimators proposed in [34,35] can be used to compare empirical data and models by generating samples from the former, the semi-empirical estimation of KL divergence and SH distance yields results with smaller variance.

This paper is organised as follows. The subsequent section provides preliminaries required for deriving the semi-empirical KL divergence and SH distance estimator. In Section 4, the semi-empirical estimation method derived in Section 3 is used to assess the goodness of fit for K and GP distributions when applied to empirical data from the commonly used IPIX dataset [36]. Additional numerical examples are provided to demonstrate that the proposed method is not limited to K and GP distributions only. These examples draw inspiration from those presented in [34,35]. Finally, a conclusion is given in Section 5.

## 2. Preliminaries

Suppose that empirical data are given as a totally ordered set of independent and identically distributed (i.i.d.) samples from an unknown univariate probability distribution *p* and are denoted as $X=\left\{x_{i},i=1,\ldots ,n\right\}$. While the CDF of *p* is denoted as *P*, the empirical CDF of set X is denoted as $P_{e}$ and is given as a sum of Heaviside unit step piecewise constant functions [37].

$$\theta \left(x\right)=\left\{\begin{array}{cc} 1, & x>0\\ 1/2, & x=0\\ 0, & x<0 \end{array}\right.$$

such that

(1) $$P_{e}\left(x\right)=\frac{1}{n}\sum_{i=1}^{n}\theta \left(x-x_{i}\right).$$

The univariate model, an approximation of *P* expressed in analytical form, has the PDF and CDF labelled as *q* and *Q*, respectively. Samples drawn from this distribution are represented as a totally ordered set $X^{\prime }=\left\{x_{j}^{\prime },j=1,\ldots ,m\right\}$.

In [34], the discrete distribution (1) is linearised as

(2) $$P_{c}\left(x\right)=\left\{\begin{array}{cc} 0, & x<x_{0}<\inf\left\{X,X^{\prime }\right\}\\ \alpha_{i}x+\beta_{i}, & x_{i-1}\leq x<x_{i},i=1,\ldots ,n\\ 1, & x\geq x_{n+1}>\sup\left\{X,X^{\prime }\right\} \end{array}\right.$$

which makes $P_{c}$ continuous. While $\alpha_{i}$ and $\beta_{i}$ are chosen to match $P_{c}$ at sample points of $P_{e}$, the values of $x_{0}$ and $x_{n+1}$ are not important.

Increments of the continuous empirical distribution $P_{c}$ and model distribution *Q* with respect to a sample $x_{i}\in X$ are defined as

$$\begin{array}{cc} \delta P_{c}\left(x_{i}\right) & =P_{c}\left(x_{i}\right)-P_{c}\left(x_{i}-\epsilon \right)\\ \delta Q(x_{i}) & =Q\left(x_{i}\right)-Q\left(x_{i}-\epsilon \right), \end{array}$$

∀ϵ$\in \left(0,\min_{i=1,\ldots ,n}\left\{x_{i}-x_{i-1}\right\}\right)$. For samples of increment $\Delta x_{i}=x_{i}-x_{i-1}$, the increment of the continuous empirical distribution is defined as

$$\Delta P_{c}\left(x_{i}\right)=P_{c}\left(x_{i}\right)-P_{c}\left(x_{i-1}\right)$$

and the increment of model distribution as

$$\Delta Q\left(x_{i}\right)=Q\left(x_{i}\right)-Q\left(x_{i-1}\right).$$

Similarly, increments of continuous empirical and model distributions with respect to a sample $x_{j}^{\prime }\in X^{\prime }$ are defined as

$$\begin{array}{cc} \delta P_{c}\left(x_{j}^{\prime }\right) & =P_{c}\left(x_{j}^{\prime }\right)-P_{c}\left(x_{j}^{\prime }-\epsilon \right),\\ \delta Q(x_{j}^{\prime }) & =Q\left(x_{j}^{\prime }\right)-Q\left(x_{j}^{\prime }-\epsilon \right). \end{array}$$

Furthermore, the definition of sample points in set X with reference to sample $x_{j}^{\prime }$ is

$$\begin{array}{cc} x_{nj}^{+} & =\min_{i=1,\ldots ,n}\left\{x_{i}:x_{i}\geq x_{j}^{\prime }\right\}\\ x_{nj}^{-} & =\max_{i=1,\ldots ,n}\left\{x_{i}:x_{i}<x_{j}^{\prime }\right\} \end{array}$$

which allows the definition of the sampling interval in set X with reference to the interval $\Delta x_{j}^{\prime }=x_{j}^{\prime }-x_{j-1}^{\prime }$ as

$$\begin{array}{c} \Delta x_{nj}=x_{nj}^{+}-x_{nj}^{-}. \end{array}$$

Thus, increments of continuous empirical and model distributions with respect to samples of increment $\Delta x_{j}^{\prime }$ are defined as

$$\begin{array}{cc} \Delta P_{c}\left(x_{nj}\right) & =P_{c}\left(x_{nj}^{+}\right)-P_{c}\left(x_{nj}^{-}\right)\\ \Delta Q(x_{j}^{\prime }) & =Q\left(x_{j}^{\prime }\right)-Q\left(x_{j-1}^{\prime }\right). \end{array}$$

In [38], a divergence between distributions *P* and *Q* is introduced as symmetrised Jeffreys distance *J* [39], employing corresponding densities *p* and *q* as

(3) $$J=\int_{R}\left(p(x)-q(x)\right)\log\left(\frac{p(x)}{q(x)}\right)dx,$$

where continuous distributions *P* and *Q* exist with respect to a Lebesgue measure. Divergence (3) can be understood as symmetric α-divergence family [40], interpreted here for univariate densities as

(4) $$D^{\left(\alpha \right)}=\frac{1}{2}\int \frac{\left(p{\left(x\right)}^{\alpha }-q{\left(x\right)}^{\alpha }\right)\left(p{\left(x\right)}^{1-\alpha }-q{\left(x\right)}^{1-\alpha }\right)}{\alpha (1-\alpha )}dx,\;\alpha \in R\setminus \left\{0,1\right\}$$

in the special limiting case where α→0 or α→1 such that $\lim_{\alpha \to 0}D^{\left(0\right)}=\lim_{\alpha \to 1}D^{\left(1\right)}=J$. Divergence (3) can be rewritten as a sum of more commonly used asymmetrical forward

(5) $$D\left(P||Q\right)=\int_{R}p\left(x\right)\log\left(\frac{p(x)}{q(x)}\right)dx$$

and reverse

(6) $$D\left(Q||P\right)=\int_{R}q\left(x\right)\log\left(\frac{q(x)}{p(x)}\right)dx$$

KL divergences as

(7) $$\begin{array}{cc} J & =\int_{R}p\left(x\right)\log\left(\frac{p(x)}{q(x)}\right)dx+\int_{R}q\left(x\right)\log\left(\frac{q(x)}{p(x)}\right)dx\\ \; & =D(P||Q)+D(Q||P),\;D(P||Q),D(Q||P)\geq 0, \end{array}$$

which are, throughout the remainder of this work, estimated using a semi-empirical method. For α=1/2, (4) simplifies to the SH distance as $D^{\left(1/2\right)}=4H^{2}$, where the SH distance

$$H^{2}=\frac{1}{2}\int_{R}{\left(\sqrt{p(x)}-\sqrt{q(x)}\right)}^{2}dx$$

between unknown distributions *P* and *Q* can be expressed with Hellinger affinity

(8) $$A=1-H^{2}=\int_{R}\sqrt{p(x)q(x)}dx,$$

utilising their corresponding densities *p* and *q*. Observe that the Hellinger affinity (8) shares the same definition as the Bhattacharyya coefficient, initially introduced in [41]. Furthermore, the value $\sqrt{1-A}$ meets the triangle inequality [42], a property that KL divergence lacks. Both the Hellinger affinity and the Bhattacharyya coefficient are interpreted as indicators of similarity between two probability distributions.

## 3. Derivation of Semi-Empirical Estimator

In this section, a semi-empirical estimator for KL divergence and SH distance between the empirical and unknown distribution *P* and a model distribution *Q* are derived.

### 3.1. Semi-Empirical KL Divergence Estimator

Drawing upon the findings from [34], the estimation of forward KL divergence D(P||Q) is proposed as

(9) $$\hat{D}\left(P||Q\right)=\frac{1}{n}\sum_{i=1}^{n}\log\left(\begin{array}{c} \frac{\delta P_{c}\left(x_{i}\right)}{\delta Q(x_{i})} \end{array}\right),$$

which is semi-empirical as $P_{c}$ is a continuous empirical distribution, given with (2), and *Q* is a model distribution in analytic form, with the approximation of *P* based on samples from set X. An analogous, semi-empirical estimation of reverse KL divergence D(Q||P) is proposed as

(10) $$\hat{D}\left(Q||P\right)=\frac{1}{m}\sum_{j=1}^{m}\log\left(\begin{array}{c} \frac{\delta Q(x_{j}^{\prime })}{\delta P_{c}\left(x_{j}^{\prime }\right)} \end{array}\right).$$

The corollary presented in the following section pertains to Theorem 1 outlined in [34] and is reproduced here for clarity of further reading.

**Theorem** **1** ([34])**.** *Let P and Q be absolutely continuous probability measures and assume its KL divergence is finite. Let $X={\left\{x_{i}\right\}}_{i=1}^{n}$ and $X^{\prime }={\left\{x_{i}^{\prime }\right\}}_{i=1}^{m}$ be i.i.d. samples sorted in increasing order, respectively, from P and Q; then,*

$$\hat{D}\left(P||Q\right)-1\overset{a.s.}{\to }D\left(P||Q\right).$$

In the proof of Theorem 1 in [34], the authors rearranged $\hat{D}\left(P||Q\right)$ as

(11) $$\hat{D}\left(P||Q\right)=\frac{1}{n}\sum_{i=1}^{n}\log\left(\frac{\frac{\Delta P(x_{i})}{\Delta x_{i}}}{\frac{\Delta Q(x_{mi}^{\prime })}{\Delta x_{mi}^{\prime }}}\right)-\frac{1}{n}\sum_{i=1}^{n}\log\left(\frac{\Delta P(x_{i})}{\Delta P_{c}\left(x_{i}\right)}\right)+\frac{1}{n}\sum_{i=1}^{n}\log\left(\frac{\Delta Q(x_{mi}^{\prime })}{\Delta Q_{c}\left(x_{mi}^{\prime }\right)}\right)$$

where $\Delta x_{mi}^{\prime }=\min\left\{x_{j}^{\prime }|x_{j}^{\prime }\geq x_{i}\right\}-\max\left\{x_{j}^{\prime }|x_{j}^{\prime }<x_{i}\right\}$ and $\Delta Q_{c}\left(x_{mi}^{\prime }\right)=Q\left(\min\left\{x_{j}^{\prime }|x_{j}^{\prime }\geq x_{i}\right\}\right)-Q(\max\left\{x_{j}^{\prime }|x_{j}^{\prime }<x_{i}\right\})$. The authors demonstrated that the first term in Equation (11) converges almost surely to D(p||q), the second term converges almost surely to the negated Euler constant −γ [43] (p. 905) and the third term converges almost surely to 1−γ. Combining these results, the authors concluded that $\hat{D}\left(P||Q\right)-1\overset{a.s.}{\to }D\left(P||Q\right)$.

**Corollary** **1.** *Let Q denote the model CDF expressed in analytic form. The set of samples drawn from this distribution is denoted as $X^{\prime }=\left\{x_{j}^{\prime },j=1,\ldots ,m\right\}$. Set $X^{\prime }$ is obtained through the inverse transformation $Q^{-1}\left(U\right)$ with U={j/(m+1),j=1,…,m} as a totally ordered set. Then,*

$$\hat{D}\left(P||Q\right)-\gamma \overset{a.s.}{\to }D\left(P||Q\right)$$

*and*

$$\hat{D}\left(Q||P\right)-1+\gamma \overset{a.s.}{\to }D\left(Q||P\right).$$

**Proof** **of Corollary 1.** Given that U forms a totally ordered set, it follows that $X^{\prime }$ also constitutes a totally ordered set. Each element $u_{j}\in U$ is the expected value of *j*-th order statistics in a sample size of *m* drawn from a uniform distribution over the open interval (0,1) [44] (p. 61). Thus, compared to the empirical method proposed in [34], stochastic realisations of set $X^{\prime }$ are replaced with deterministic values, which reduces the estimation variance. Furthermore, as $P_{c}$ is continuous, (9) can be rewritten as in [34]:

(12) $$\hat{D}\left(P||Q\right)=\frac{1}{n}\sum_{i=1}^{n}\log\left(\frac{\frac{\Delta P_{c}\left(x_{i}\right)}{\Delta x_{i}}}{\frac{\Delta Q(x_{i})}{\Delta x_{i}}}\right),$$

and further reformulated as

(13) $$\hat{D}\left(P||Q\right)=\frac{1}{n}\sum_{i=1}^{n}\log\left(\frac{\frac{\Delta P(x_{i})}{\Delta x_{i}}}{\frac{\Delta Q(x_{i})}{\Delta x_{i}}}\right)-\frac{1}{n}\sum_{i=1}^{n}\log\left(\frac{\Delta P(x_{i})}{\Delta P_{c}\left(x_{i}\right)}\right).$$

The first term of the right-hand side converges almost surely to D(P||Q), and the second results in $\frac{1}{n}\sum_{i=1}^{n}\log\left(n\Delta P(x_{i})\right)$. As indicated in [34], if realisation $P(x_{i})$ is thought of as a time event at $x_{i}$ in a Poisson point process, then $\Delta P\left(x_{i}\right)=P\left(x_{i}\right)-P\left(x_{i-1}\right)$ is the time difference between two successive events, and quantity $z_{i}=n\Delta P\left(x_{i}\right)$ follows an exponential distribution f(z;1)=exp(−z) with a mean of 1. Thus,

$$\frac{1}{n}\sum_{i=1}^{n}\log\left(z_{i}\right)\overset{a.s.}{\to }\int_{R}\log\left(z\right)f\left(z;1\right)dz=\int_{0}^{\infty }\log\left(z\right)\exp\left(-z\right)dz$$

where $\int_{0}^{\infty }\log\left(z\right)\exp\left(-z\right)dz=-\gamma$ [43] (p. 906) and (13) converges almost surely to D(P||Q)+γ.

Semi-empirical estimation given with (10) can be further reformulated to

(14) $$\hat{D}\left(Q||P\right)=\frac{1}{m}\sum_{j=1}^{m}\log\left(\frac{\frac{\Delta Q(x_{j}^{\prime })}{\Delta x_{j}^{\prime }}}{\frac{\Delta P(x_{nj})}{\Delta x_{nj}}}\right)+\frac{1}{m}\sum_{j=1}^{m}\log\left(\frac{\Delta P(x_{nj})}{\Delta P_{c}\left(x_{nj}\right)}\right).$$

The first term of the right-hand side converges almost surely to D(Q||P), and, as demonstrated in [34], the summation in the second term can be rephrased as the sum of samples from set X, incorporating a multiplication factor $m\Delta Q(x_{i})$. This factor represents the number of samples from set $X^{\prime }$ occurring between two consecutive samples from set X. Thus,

(15) $$\begin{array}{cc} \frac{1}{m}\sum_{j=1}^{m}\log\left(\frac{\Delta P(x_{nj})}{\Delta P_{c}\left(x_{nj}\right)}\right)\; & =\frac{1}{m}\sum_{i=1}^{n}m\Delta Q\left(x_{i}\right)\log\left(\frac{\Delta P(x_{i})}{\Delta P_{c}\left(x_{i}\right)}\right)\\ \; & =\frac{1}{n}\sum_{i=1}^{n}\frac{\frac{\Delta Q(x_{i})}{\Delta x_{i}}}{\frac{\Delta P(x_{i})}{\Delta x_{i}}}n\Delta P\left(x_{i}\right)\log\left(n\Delta P(x_{i})\right). \end{array}$$

As before, $\Delta P(x_{i})$ can be thought of as a time difference between two consecutive events at $x_{i}$ and $x_{i-1}$ in a Poisson point process, so $z_{i}=n\Delta P\left(x_{i}\right)$ follows an exponential distribution f(z;1)=exp(−z) with a mean value of 1. Moreover, as $\Delta Q\left(x_{i}\right)/\Delta x_{i}$ approaches q(x) and $\Delta P\left(x_{i}\right)/\Delta x_{i}$ approaches p(x),

$$\begin{array}{cc} \frac{1}{m}\sum_{j=1}^{m}\log\left(\frac{\Delta P(x_{nj})}{\Delta P_{c}\left(x_{nj}\right)}\right)\; & \overset{a.s.}{\to }\int_{R^{2}}p\left(x\right)\frac{q(x)}{p(x)}z\log\left(z\right)f\left(z;1\right)dxdz\\ \; & =\int_{R}q\left(x\right)dx\int_{0}^{\infty }z\log\left(z\right)\exp\left(-z\right)dz. \end{array}$$

Since $\int_{R}q\left(x\right)dx=1$ and $\int_{0}^{\infty }z\log\left(z\right)\exp\left(-z\right)dz$ results in 1−γ [43] (p. 573), (14) converges almost surely to D(Q||P)+1−γ. □

### 3.2. Semi-Parametric SH Distance Estimator

Building upon a finding from [35], the estimation of (8) involves employing a continuous empirical distribution $P_{c}$ and a model distribution *Q* expressed in analytic form, as proposed in the following manner:

(16) $$\hat{A}\left(P,Q\right)=\frac{1}{n}\sum_{i=1}^{n}\sqrt{\frac{\delta Q(x_{i})}{\delta P_{c}\left(x_{i}\right)}},$$

which is a semi-empirical estimation, as argued in the previous subsection. To resemble the notation used for the estimation of KL divergence in the previous subsection, (16) is referred to as forward estimation. Given the symmetry of the SH distance, the reverse estimation of Hellinger affinity is proposed as

(17) $$\hat{A}\left(Q,P\right)=\frac{1}{m}\sum_{j=1}^{m}\sqrt{\frac{\delta P_{c}\left(x_{j}^{\prime }\right)}{\delta Q(x_{j}^{\prime })}},$$

and a semi-empirical estimator of SH distance is denoted as

(18) $$\hat{H^{2}}=1-\hat{A},\;\hat{A}=\frac{1}{2}\left(\hat{A}\left(P,Q\right)+\hat{A}\left(Q,P\right)\right)$$

which is similar to the approach taken by the authors in [35]. Throughout the remainder of this subsection, it will be demonstrated that semi-empirical estimates (16) and (17) converge almost surely to the true value of *A*. Therefore, the following corollary is derived from Theorem 1 [34], as well as insights from [35].

**Corollary** **2.** *Let Q denote the model CDF expressed in analytic form. The set of samples drawn from this distribution is denoted as $X^{\prime }=\left\{x_{j}^{\prime },j=1,\ldots ,m\right\}$. Set $X^{\prime }$ is obtained through the inverse transformation $Q^{-1}\left(U\right)$ with U={j/(m+1),j=1,…,m} as a totally ordered set. Then,*

$$\begin{array}{cc} \frac{2}{\sqrt{\pi }}\hat{A}\left(P,Q\right) & \overset{a.s.}{\to }A,\\ \frac{2}{\sqrt{\pi }}\hat{A}\left(Q,P\right) & \overset{a.s.}{\to }A, \end{array}$$

*and consequently,*

$$\frac{4}{\sqrt{\pi }}\hat{A}\overset{a.s.}{\to }A.$$

**Proof** **Corollary 2.** Applying the identical reasoning as presented in the proof of Corollary 1, (16) is rewritten as

$$\hat{A}\left(P,Q\right)=\frac{1}{n}\sum_{i=1}^{n}\sqrt{\frac{\frac{\Delta Q(x_{i})}{\Delta x_{i}}}{\frac{\Delta P(x_{i})}{\Delta x_{i}}}}\sqrt{\frac{\Delta P(x_{i})}{\Delta P_{c}\left(x_{i}\right)}}=\frac{1}{n}\sum_{i=1}^{n}\sqrt{\frac{\frac{\Delta Q(x_{i})}{\Delta x_{i}}}{\frac{\Delta P(x_{i})}{\Delta x_{i}}}}\sqrt{z},$$

where $z=n\Delta P(x_{i})$ follows a unit-mean exponential distribution f(z;1)=exp(−z). Therefore, as $\Delta Q\left(x_{i}\right)/\Delta x_{i}$ tends to q(x) and $\Delta P\left(x_{i}\right)/\Delta x_{i}$ tends to p(x), it follows that

$$\begin{array}{cc} \hat{A}\left(P,Q\right) & \overset{a.s.}{\to }\int_{R^{2}}p\left(x\right)\sqrt{\frac{q(x)}{p(x)}}\sqrt{z}f\left(z;1\right)dxdz\\ \; & =\int_{R}\sqrt{p(x)q(x)}dx\int_{0}^{\infty }\sqrt{z}\exp\left(-z\right)dz=\frac{\sqrt{\pi }}{2}A, \end{array}$$

since $\int_{0}^{\infty }\sqrt{z}\exp\left(-z\right)dz=\Gamma \left(3/2\right)=\sqrt{\pi }/2$ [43] (p. 897) and $\int_{R}\sqrt{p(x)q(x)}dx=A$ with the help of the Hellinger affinity definition (8).

The reverse estimation of Hellinger affinity (17) can be reformulated as

$$\hat{A}\left(Q,P\right)=\frac{1}{m}\sum_{j=1}^{m}\sqrt{\frac{\frac{\Delta P(x_{nj})}{\Delta x_{nj}}}{\frac{\Delta Q(x_{j}^{\prime })}{\Delta x_{j}^{\prime }}}}\sqrt{\frac{\Delta P_{c}\left(x_{nj}\right)}{\Delta P(x_{nj})}}=\frac{1}{m}\sum_{j=1}^{m}\sqrt{\frac{\frac{\Delta P(x_{nj})}{\Delta x_{nj}}}{\frac{\Delta Q(x_{j}^{\prime })}{\Delta x_{j}^{\prime }}}}\frac{1}{\sqrt{n\Delta P(x_{nj})}},$$

and considering that $n\Delta P(x_{nj})$ is independent from the distribution *P*,

$$\hat{A}\left(Q,P\right)\overset{a.s.}{\to }\left(\frac{1}{m}\sum_{j=1}^{m}\sqrt{\frac{\frac{\Delta P(x_{nj})}{\Delta x_{nj}}}{\frac{\Delta Q(x_{j}^{\prime })}{\Delta x_{j}^{\prime }}}}\right)\left(\frac{1}{m}\sum_{j=1}^{m}\frac{1}{\sqrt{n\Delta P(x_{nj})}}\right).$$

While the first sum of the right-hand side converges almost surely to *A* when the ratios $\Delta P\left(x_{nj}\right)/\Delta x_{nj}$ and $\Delta Q\left(x_{j}^{\prime }\right)/\Delta x_{j}^{\prime }$ tend to p(x) and q(x), respectively, the second sum can be restated by utilising samples from the set X and incorporating the factor $m\Delta Q(x_{i})$. Here, as previously mentioned in Section 3.1 and illustrated in (15), $m\Delta Q(x_{i})$ denotes the count of samples from set $X^{\prime }$ between two consecutive samples from set X. Thus,

(19) $$\frac{1}{m}\sum_{j=1}^{m}\frac{1}{\sqrt{n\Delta P(x_{nj})}}=\frac{1}{m}\sum_{i=1}^{n}\frac{m\Delta Q(x_{i})}{\sqrt{n\Delta P(x_{i})}}=\frac{1}{n}\sum_{i=1}^{n}\frac{\frac{\Delta Q(x_{i})}{\Delta x_{i}}}{\frac{\Delta P(x_{i})}{\Delta x_{i}}}\frac{n\Delta P(x_{i})}{\sqrt{n\Delta P(x_{i})}}.$$

From the findings outlined in Section 3.1, it is evident that the variable $z_{i}=n\Delta P\left(x_{i}\right)$ follows a unit-mean exponential distribution f(z;1)=exp(−z), so from (19), it follows that

$$\begin{array}{cc} \frac{1}{m}\sum_{j=1}^{m}\frac{1}{\sqrt{n\Delta P(x_{nj})}}\; & \overset{a.s.}{\to }\int_{R^{2}}p\left(x\right)\frac{q(x)}{p(x)}\sqrt{z}f\left(z;1\right)dxdz\\ \; & =\int_{R}q\left(x\right)dx\int_{0}^{\infty }\sqrt{z}\exp\left(-z\right)dz=\frac{\sqrt{\pi }}{2}, \end{array}$$

since $\int_{R}q\left(x\right)dx$ is 1 and $\int_{0}^{\infty }\sqrt{z}\exp\left(-z\right)dz=\Gamma \left(3/2\right)=\sqrt{\pi }/2$. Therefore, since $2\hat{A}\left(P,Q\right)/\sqrt{\pi }$ and $2\hat{A}\left(Q,P\right)/\sqrt{\pi }$ converge almost surely to *A*, from (18), it follows that $4\hat{A}/\sqrt{\pi }$ also converges almost surely to *A*. □

## 4. Numerical Examples

The following subsection focuses on evaluating the goodness of fit for some commonly used radar sea clutter models. The subsequent subsection explores additional examples unrelated to sea clutter models, highlighting instances where convergence limitations may be observed, but also presenting an instance where convergence is reached with a moderate number of samples.

### 4.1. Radar Sea Clutter

Within this subsection, two illustrative examples showing the performance of the proposed semi-empirical estimators are given. The presented results are compared with those derived from empirical estimators proposed in [34,35]. Both the semi-empirical KL divergences (9) and (10), along with the SH distance (18), are included. The presented examples cover a scenario where the true distribution is assumed to be known, as well as a situation where it remains unknown. Examples draw inspiration from [45], where the authors chose to fit specific datasets from a measurement campaign [36] without specifying which datasets were used. Upon examining the available files from [36], it was found that datasets 17 and 31 were utilised. In both cases, cell number 14 was tested. As a remark, selected datasets are particularly interesting as they share some properties found in COTS radar sensors and correspond to significant wave heights of 2.3m (dataset 17) and 0.9m (dataset 31) which represent two-thirds of the total wave heights observed in the Adriatic Sea [46]. The fitting involved K and GP distributions as models. A brief overview of these distributions is provided in Appendix A.

In the first example from [45], a heavy-tailed distribution is observed and the GP distribution provides a good fit to the data. Therefore, it is treated as the known distribution, and KL divergences and the SH distance between this distribution and the K distribution as a model are estimated. However, in the second example from [45], a distribution with a moderate tail is observed and it remains uncertain which distribution better captures the data, K or GP. Consequently, KL divergences and SH distance are estimated using actual data, with the K and GP distributions as models.

To evaluate the KL divergence and SH distance using data from the first example in [45], the model distribution *P* is defined as

(20) $$P\left(x\right)=c_{p}\left(1-\frac{4^{\nu_{p}}}{{\left(\beta_{p}x^{2}+4\right)}^{\nu_{p}}}\right),\;\nu_{p},\beta_{p}>0,0\leq x\leq x_{m}$$

for which clutter amplitude intensity $z=x^{2}$ is GP distributed. Truncated distribution (20) has shape and scale parameters denoted as $\nu_{p}=1.02$ and $\beta_{p}=49678.19\;V^{-2}$, respectively. Constant $c_{p}$ depends on the maximum observed amplitude value $x_{m}$ as

$$c_{p}^{-1}=1-\frac{4^{\nu_{p}}}{{\left(\beta_{p}x_{m}^{2}+4\right)}^{\nu_{p}}}$$

which is, from the results presented in [45], approximately 0.35V. Likewise, the model distribution *Q* is defined as a truncated K-distribution CDF

(21) $$Q\left(x\right)=c_{q}\left(1-\frac{2x^{\nu_{q}}}{\sqrt{\beta_{q}^{\nu_{q}}}\Gamma \left(\nu_{q}\right)}K_{\nu_{q}}\left(\frac{2x}{\sqrt{\beta_{q}}}\right)\right),\;\nu_{q},\beta_{q}>0,0\leq x\leq x_{m},$$

with the shape parameter set to $\nu_{q}=0.38$ and the scale parameter set to $\beta_{q}=0.00109\;V^{2}$, for $0\leq x\leq x_{m}$. In (21), *K* denotes a modified Bessel function of the second kind [47], and the constant $c_{q}$ also depends on $x_{m}$ as

$$c_{q}^{-1}=1-\frac{2x_{m}^{\nu_{q}}}{\sqrt{\beta_{q}^{\nu_{q}}}\Gamma \left(\nu_{q}\right)}K_{\nu_{q}}\left(\frac{2x_{m}}{\sqrt{\beta_{q}}}\right).$$

Truncating the distributions (20) and (21) prevents numerical underflow in the denominator of (9). This issue arises particularly with high-amplitude values ($x\gg x_{m}$), which possess a notable probability of occurrence due to the heavy-tailed nature of (20). In other words, when the numerator in Equation (9) follows a heavy-tailed distribution (e.g., GP distribution) and the denominator follows a moderate-tailed distribution (e.g., K distribution), large sample amplitudes from the heavy-tailed empirical data can cause the denominator to reduce to zero, resulting in numerical underflow. Truncating the heavy-tailed distribution helps prevent such occurrences.

The numerical experiment is organised as follows. In order to reproduce the empirical estimation proposed in [34,35], two equally sized sets are generated from densities *p* and *q*, corresponding to distributions *P* and *Q*, respectively. Specifically, $X=\{x_{i}∼p\left(\nu_{p},\beta_{p},x_{m}\right),i=1,\ldots ,n\}$ and $X^{\prime }=\left\{x_{j}^{\prime }∼q\left(\nu_{q},\beta_{q},x_{m}\right),j=1,\ldots ,m=n\right\}$. Their respective empirical CDFs are then used as inputs for the empirical estimators. For the semi-empirical estimation, set X is kept as is and set $X^{\prime }$ is constructed with the help of inverse transformation $X^{\prime }=Q^{-1}\left(U\right)$. Here, the set U={j/(m+1),j=1,…,m} is a totally ordered set as described in the proof of Corollary 1, and *Q* is defined by (21). Generated samples X and $X^{\prime }$ were subjected to the KS test to determine whether they followed truncated K (A5) and GP (A6) distributions. The KS test [48] validated the null hypothesis $H_{0}$ that the samples are drawn from truncated K and GP distributions, indicating finite KS distances. Additionally, the Ljung–Box test for independence [49] was performed, and the null hypothesis $H_{0}$ that the samples are independently distributed was also accepted. The Monte Carlo method for simulating samples X and $X^{\prime }$ is described in greater detail in Appendix B and Table A1.

**Figure 1:**
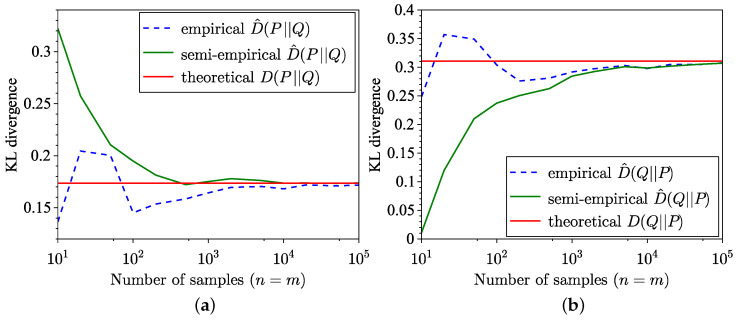
Comparison of empirical and semi-empirical estimates of KL divergence. (**a**) Forward. (**b**) Reverse.

**Figure 2:**
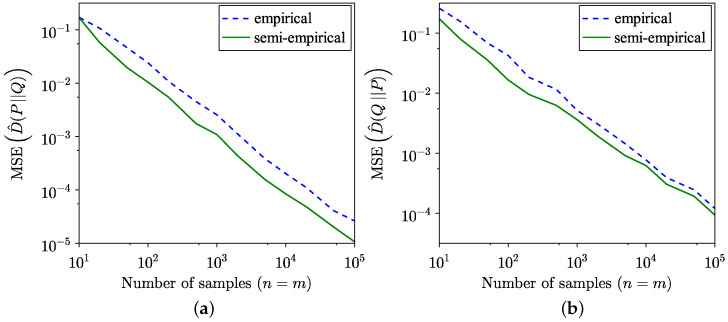
Comparison of MSE of empirical and semi-empirical estimates of KL divergence. (**a**) Forward. (**b**) Reverse.

The comparison between the KL divergence estimates across 100 trials using the empirical method from [34] and the semi-empirical methods (9) and (10) is illustrated in Figure 1. Although the semi-empirical KL divergence estimation demonstrates lower MSE, as highlighted in Figure 2, it exhibits a slower convergence to the true value for the specific distributions considered in this example. The MSE is defined for forward estimation as

(22) $$\mathrm{MSE}\left(\hat{D}\left(P||Q\right)\right)=\frac{1}{n}\sum_{i=1}^{n}{\left({\hat{D}}_{i}\left(P||Q\right)-D\left(P||Q\right)\right)}^{2}$$

and for reverse estimation as

(23) $$\mathrm{MSE}\left(\hat{D}\left(Q||P\right)\right)=\frac{1}{n}\sum_{i=1}^{n}{\left({\hat{D}}_{i}\left(Q||P\right)-D\left(Q||P\right)\right)}^{2}$$

where the subscript *i* represents the *i*-th realisation.

**Figure 3:**
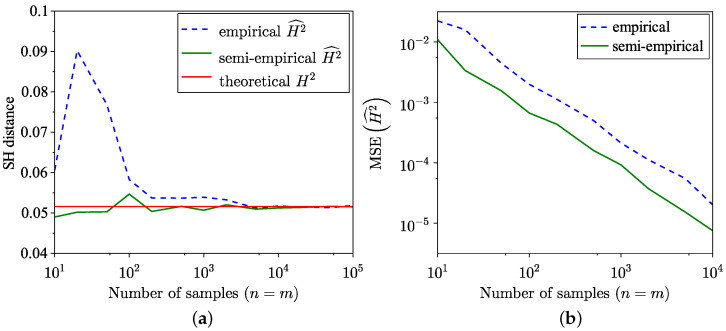
Comparison of empirical and semi-empirical estimates. (**a**) SH distance estimation. (**b**) MSE of SH distance estimation.

The findings of the SH distance estimation, utilising both the empirical method from [35] and the semi-empirical method (18) across 100 trials, are presented in Figure 3a. The MSE corresponding to these estimations is presented in Figure 3b, and, as expected, the MSE of the semi-empirical estimate is lower. Notably, for the distributions examined in this example, the semi-empirical estimation exhibits a faster convergence towards the true value. The MSE for SH distance is defined as

(24) $$\mathrm{MSE}\left(\hat{H^{2}}\right)=\frac{1}{n}\sum_{i=1}^{n}{\left({\hat{H^{2}}}_{i}-H^{2}\right)}^{2}$$

where subscript *i* stands for the *i*-th realisation.

For the second example from [45], amplitude measurements in dataset 31 are permuted to meet the condition of independent samples required by Theorem 1. Although this does not guarantee sample independence, independence will be assumed for the purposes of this example. Furthermore, amplitude in the utilised dataset was quantised in 256 levels, which makes the quantisation effect significant and, as a consequence, the condition of continuous distribution required by Theorem 1 is not met. Therefore, band-limited noise that follows the density

(25) $$w\left(x\right)=\frac{1}{c_{w}}{\left(\frac{\sin\left(\frac{\pi x}{4\Delta x}\right)}{\left(\frac{\pi x}{4\Delta x}\right)}\right)}^{4},\;c_{w}=\int_{-\infty }^{\infty }{\left(\frac{\sin\left(\frac{\pi x}{4\Delta x}\right)}{\left(\frac{\pi x}{4\Delta x}\right)}\right)}^{4}dx$$

(where Δx is the quantisation interval) was added to the amplitude samples to reconstruct the original continuous signal data [50]. Since the original data before sampling were likely not dithered, this introduces some distortion of the original density. However, visual inspection of the histogram before and after noise addition shows no significant deviation. Consequently, for the purpose of this example, the distortion is considered negligible.

Considering the aforementioned points, the numerical example is organised as follows. The dataset 13 from [36] is divided into *k* groups, each containing *n* samples, ensuring that the product kn equals 131,072, which is the total number of amplitude samples in the dataset. Each group is treated as a separate trial. The amplitude distribution of the samples is fitted using the GP distribution and the K distribution, with the parameters taken from [45]. For the GP distribution, the parameters are $\nu_{p}=1.53$ and $\beta_{p}=1067.66\;V^{-2}$, while for the K distribution, the parameters are $\nu_{q}=1$ and $\beta_{q}=0.00620\;V^{2}$. Real data involved in this example do not exhibit the high-level excursions observed in the simulated data. Therefore, the truncation of the distributions (20) and (21) is set to the maximum observed amplitude $x_{m}=0.7\;V$.

**Figure 4:**
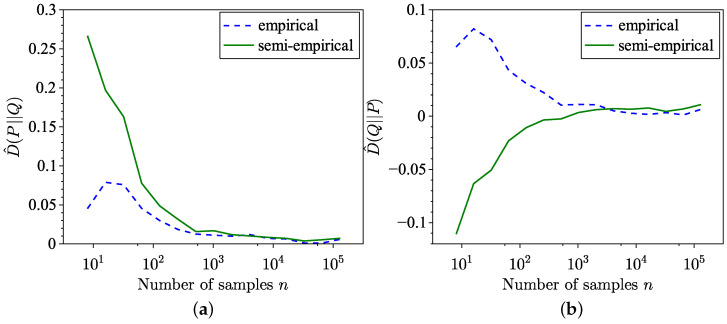
Comparison of empirical and semi-empirical estimates of KL divergence using GP distribution as model and real sea clutter data. (**a**) Forward. (**b**) Reverse.

**Figure 5:**
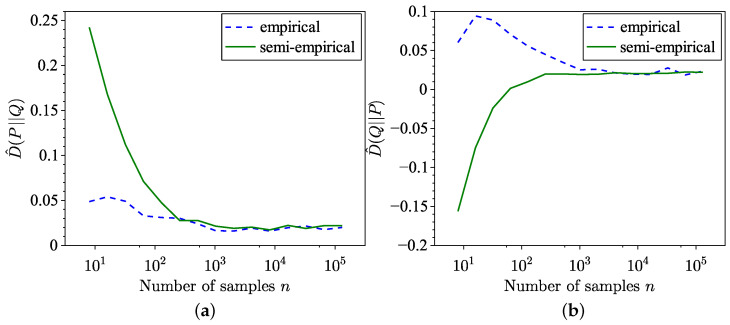
Comparison of empirical and semi-empirical estimates of KL divergence using K distribution as model and real sea clutter data. (**a**) Forward. (**b**) Reverse.

**Figure 6:**
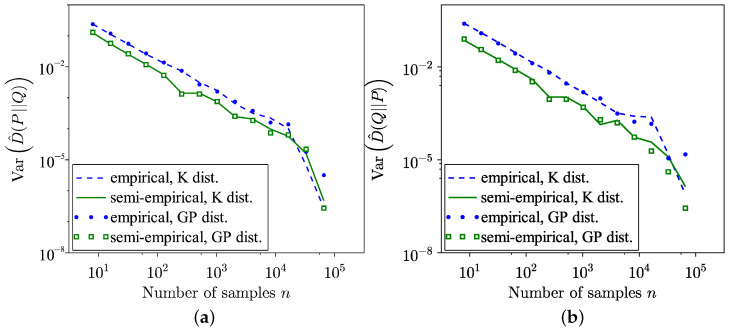
Comparison of variances of empirical and semi-empirical estimates of KL divergence using GP and K distribution as models and real sea clutter data. (**a**) Forward. (**b**) Reverse.

Figure 4 presents the results of both empirical and semi-empirical estimations of forward and reverse KL divergence for the GP distribution. Similarly, Figure 5 displays these results for the K distribution. The convergence value meets expectations, as the KL divergence between these models is 0.032 for the forward divergence and 0.03 for the reverse divergence. Given that the data in the tail region lie between values of these two models, it is reasonable to expect KL divergences to be lower than the mentioned values. Furthermore, as shown in Figure 6, although the empirical estimation converges faster, it exhibits greater estimation variance compared to the semi-empirical estimation. This observation holds true for both the GP and the K-distribution models. The difference in estimation variance between the GP and K-distribution models is not significant.

**Figure 7:**
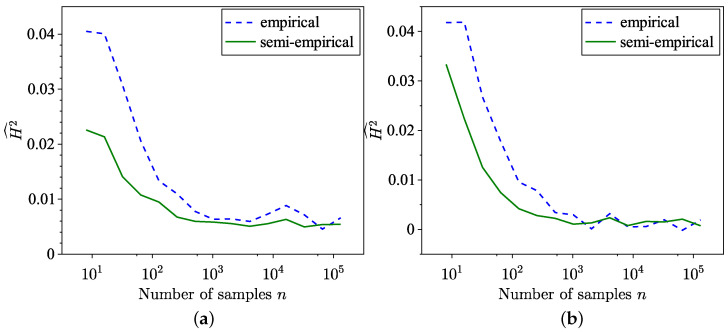
Comparison of empirical and semi-empirical estimates of SH distance using GP and K distribution as models and real sea clutter data. (**a**) K distribution. (**b**) GP distribution.

**Figure 8:**
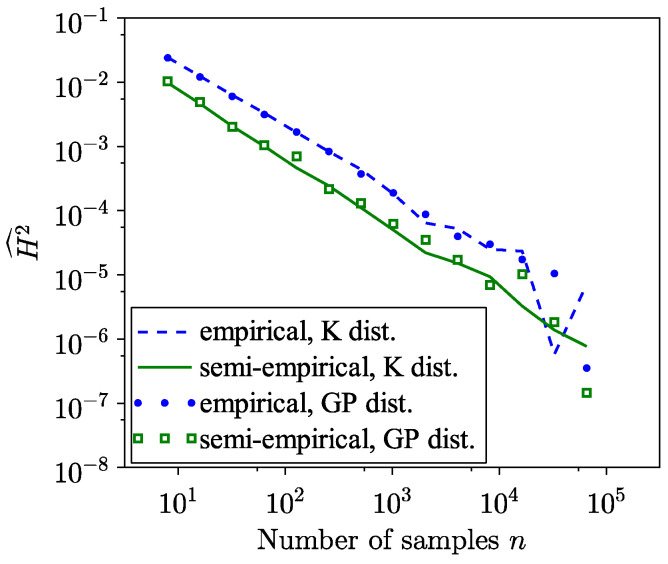
Comparison of variances of empirical and semi-empirical estimates of SH distance using GP and K distributions as models and real sea clutter data.

Regarding the SH distance estimate, Figure 6 and Figure 7 demonstrate faster convergence with a smaller variance, Figure 8, using the semi-empirical method. Given that the theoretical SH distance between models (20) and (21) is 0.0075, the observed convergence value aligns with expectations. Since the data in the tail region fall within the tails of the distributions used, it is reasonable to expect an SH distance smaller than this value. 

### 4.2. Additional Numerical Examples

**Figure 9:**
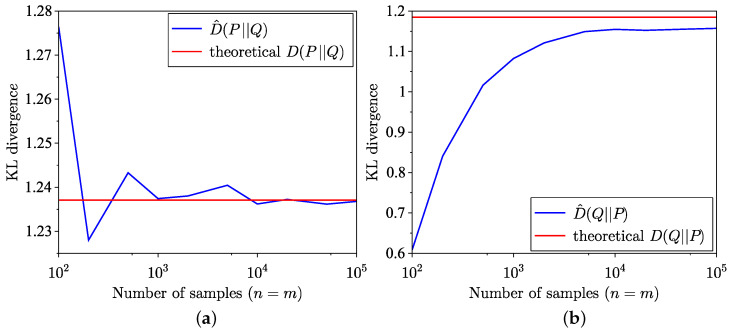
Semi-empirical estimation of KL divergence between an empirical dataset following a unit-mean exponential distribution, Exp(1), and a model distribution following a normal distribution, N(3,4). (**a**) Forward estimation. (**b**) Reverse estimation.

**Figure 10:**
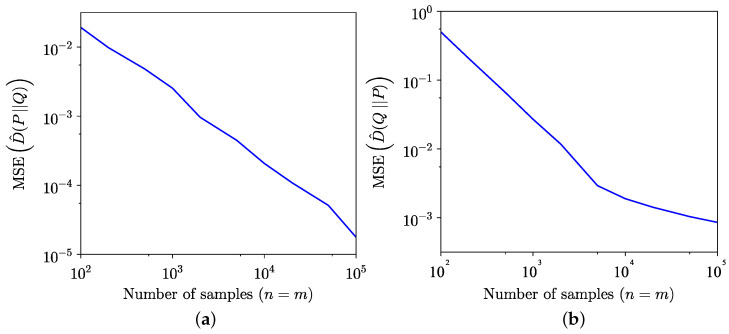
MSE of the KL divergence estimation between an empirical dataset following a unit-mean exponential distribution, Exp(1), and a model distribution following a normal distribution, N(3,4). (**a**) Forward. (**b**) Reverse.

This subsection provides additional numerical examples illustrating the semi-empirical estimation of KL divergence and SH distance, demonstrating that the proposed method works well for distributions not necessarily related to sea clutter. These examples are inspired by the numerical experiments in [34,35]. In the first part of these numerical experiments, KL divergence and SH distance are evaluated for distributions with different supports. Specifically, the KL divergence is computed between samples from a unit-mean exponential distribution, $X=\{x_{i}:x_{i}∼\mathrm{Exp}\left(1\right),i=1,\ldots ,n\}$, with support x∈[0,∞), and a model represented by a normal distribution N(3,4) with support $x^{\prime }\in R$. Forward estimation results for KL divergence are depicted in Figure 9a, while reverse estimation results are shown in Figure 9b. Notably, reverse estimation is significantly slower than forward estimation in this case, with higher MSE. A similar trend is observed for sea clutter distributions in Figure 1, where the set X is sampled from a heavy-tailed GP distribution, and the model distribution is a moderately tailed K distribution. The MSE for both forward and reverse estimations is presented in Figure 10.

**Figure 11:**
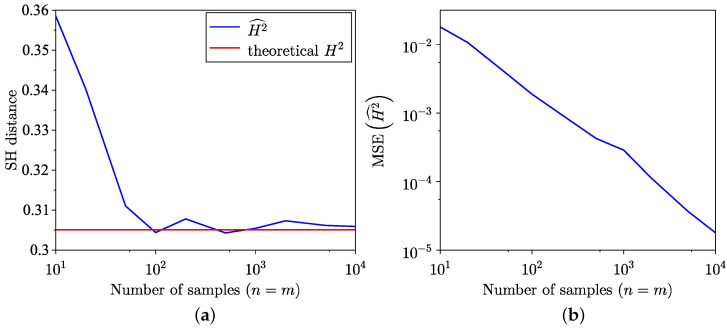
Semi-empirical estimation of SH distance between empirical dataset of samples from normal distribution N(3,4) and exponential model distribution Exp(1). (**a**) SH distance estimation. (**b**) MSE of SH distance estimation.

Unlike KL divergence estimation, SH distance estimation for distributions with non-equal support demonstrates greater robustness. This is illustrated in Figure 11a, where the samples are drawn from normal distribution $X=\{x_{i}:x_{i}∼N\left(3,2\right)\}$ and the model distribution is a unit-mean exponential. A similar pattern is observed for SH distance estimation between samples drawn from a heavy-tailed GP distribution and a model given by a K distribution, as shown in Figure 3a. The MSE for SH distance estimation is shown in Figure 11b.

**Figure 12:**
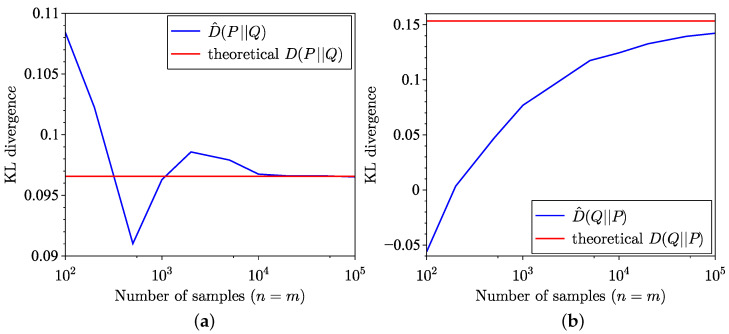
Semi-empirical estimation of the KL divergence between two normal distributions, with the empirical dataset following N(0,1) and the model distribution following N(0,2). (**a**) Forward estimation. (**b**) Reverse estimation.

**Figure 13:**
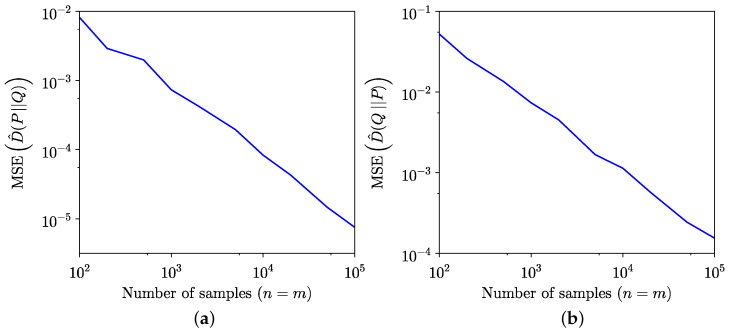
MSE of KL divergence estimation between two normal distributions, empirical dataset following N(0,1) and model distribution following N(0,2). (**a**) Forward. (**b**) Reverse.

**Figure 14:**
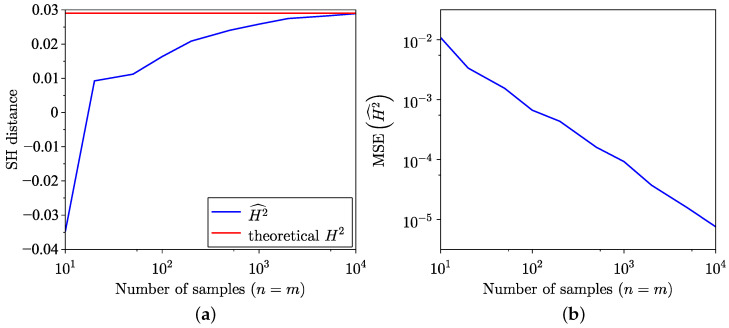
Semi-empirical estimation of SH distance between empirical dataset of samples from normal distribution N(0,1) and normal model distribution N(0,2). (**a**) SH distance estimation. (**b**) MSE of SH distance estimation.

The remainder of this subsection focuses on comparing divergences and distances between normal distributions with different parameters. Thus, Figure 12 and Figure 13 depict the KL divergence between samples drawn from zero-mean normal distributions, where the empirical distribution has a variance of 1, and the model distribution has a variance of 2. The corresponding MSE is shown in Figure 13. Notice the significantly slower convergence in the reverse estimation, leading to a higher MSE. For comparison, the SH distance between the same datasets is presented in Figure 14a, with the associated MSE provided in Figure 14b.

**Figure 15:**
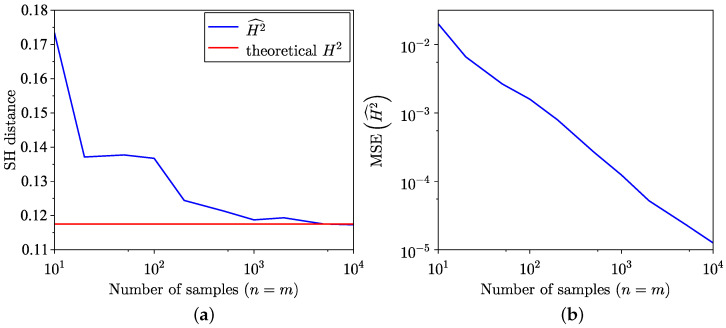
Semi-empirical estimation of SH distance between empirical dataset of samples from normal distribution N(0,1) and normal model distribution N(1,1). (**a**) SH distance estimation. (**b**) MSE of SH distance estimation.

**Figure 16:**
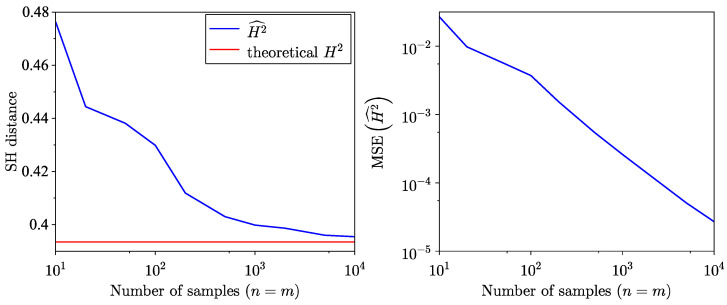
Semi-empirical estimation of SH distance between empirical dataset of samples from normal distribution N(0,1) and normal model distribution N(2,1). (**a**) SH distance estimation. (**b**) MSE of SH distance estimation.

Next, SH distances and their corresponding MSEs are compared for samples drawn from the normal distribution N(0,1) against model distributions N(1,1) and N(2,1). The results are shown in Figure 15 and Figure 16, respectively.

**Figure 17:**
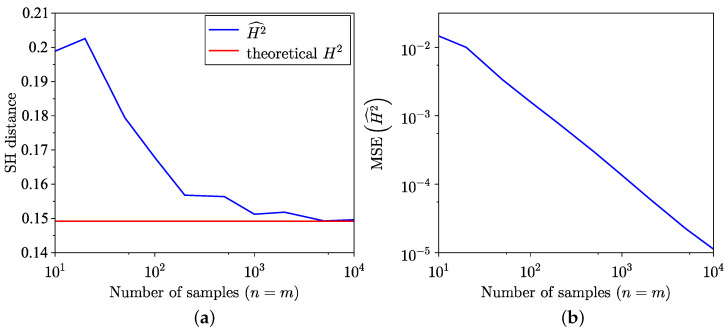
Semi-empirical estimation of SH distance between empirical dataset of samples from normal distribution N(0,4) and normal model distribution N(1,1). (**a**) SH distance estimation. (**b**) MSE of SH distance estimation.

Finally, the SH distance is estimated for samples drawn from the normal distribution N(0,4) and a model following the normal distribution N(1,1). The results are presented in Figure 17a for the estimation and Figure 17b for the MSE.

## 5. Conclusions

The suggested approach for evaluating the goodness of fit of model distributions to clutter data is part of a broader framework focused on replicating sea clutter data obtained from measurements. Such a replication is valuable for evaluating the performance of various processes, such as target-tracking algorithms, or other types of processing aimed to mitigate the effects of sea clutter returns. The results demonstrated in the examples for the representative K and GP distributions indicate that a sample size greater than 1000 is sufficient for the semi-empirical estimate to converge to the true value. Additional examples demonstrate that the proposed method is not limited to compound distributions like K and GP. In most cases, the method performs effectively, and compared to its empirical counterpart, it achieves a lower MSE because randomness originates solely from a single source, the empirical dataset. However, in certain scenarios, particularly when the supports of the sample and model distributions differ, reverse KL divergence estimation may require an extremely large sample size, exceeding 100,000, to converge. In such cases, the MSE is significantly higher compared to forward estimation.

## Data Availability

The original data presented in the study are openly available in the McMaster IPIX radar sea clutter database at http://soma.ece.mcmaster.ca/ipix/dartmouth/datasets.html (accessed on 27 September 2023).

## Abbreviations

The following abbreviations are used in this manuscript:

CDF	Cumulative Distribution Function
CGIG	Compound Gaussian Inverse Gaussian
COTS	Commercial Of the Shelf
GP	Generalised Pareto
i.i.d.	independent and identically distributed
IPIX	Intelligent PIxel processing X-band
KL	Kullback-Leibler
KS	Kolmogorov-Smirnov
MSE	Mean Square Error
PDF	Probability Distribution Function
RIB	Rigid Inflatable Boat
RiIG	Ricean Inverse Gaussian
SH	Squared Hellinger

## Appendix A

Both K and GP distributions belong to the class of complex and elliptically symmetric distributions [7] and can be observed in the context of the surface wave model of the sea, primarily composed of two components: large gravitational and smaller capillary waves, as described in [51]. Consequently, the complex envelope of clutter returns can be written as $d=x\sqrt{\zeta }$ [52], where x denotes a speckle component that is the result of reflections from a large number of uniformly distributed reflectors within the sensor’s resolution cell, resulting in a complex Gaussian process with a temporal correlation of several milliseconds, and ζ is due to the reflections of electromagnetic energy from large gravitational (wind-driven and swell) waves and it describes the background power with a temporal correlation of several seconds. If the variances of the in-phase and quadrature-phase components of x are normalised to 1 as in [45] and ζ is gamma distributed with shape and scale parameters $\nu_{q}$ and $\beta_{q}$, defined as

(A1) $$p\left(\zeta \right)=\frac{\zeta^{\nu_{q}-1}}{{\left(\frac{\beta_{q}}{2}\right)}^{\nu_{q}}\Gamma \left(\nu_{q}\right)}\exp\left(-\frac{2\zeta }{\beta_{q}}\right),\;\zeta ,\nu_{q},\beta_{q}>0,$$

then the amplitude distribution q(x=|d|) follows a K distribution

(A2) $$q\left(x=|d|\right)=\frac{4x^{\nu_{q}}}{\beta_{q}^{\frac{\nu_{q}+1}{2}}\Gamma \left(\nu_{q}\right)}K_{\nu_{q}-1}\left(\frac{2x}{\sqrt{\beta_{q}}}\right),\;x\geq 0$$

where Γ is the gamma function and *K* denotes the modified Bessel function of the second kind [47]. Moreover, if ζ is distributed as inverse gamma with shape and scale parameters $\nu_{p}$ and $\beta_{p}$, given by

(A3) $$p\left(\zeta \right)=\frac{\zeta^{-\nu_{p}-1}}{{\left(\frac{\beta_{p}}{2}\right)}^{\nu_{p}}\Gamma \left(\nu_{p}\right)}\exp\left(-\frac{2}{\beta_{p}\zeta }\right),\;\zeta ,\nu_{p},\beta_{p}>0,$$

the amplitude distribution p(x=|d|) is given by

(A4) $$p\left(x=|d|\right)=\frac{x\beta_{p}\Gamma \left(\nu_{p}+1\right)}{2{\left(\frac{\beta_{p}}{4}x^{2}+1\right)}^{\nu_{p}+1}\Gamma \left(\nu_{p}\right)},\;x\geq 0$$

which corresponds to a GP distribution for the amplitude intensity $z=x^{2}$. Truncated versions of (A2) and (A4) are given as

(A5) $$q\left(x=|d|\right)={\left(1-\frac{2x_{m}^{\nu_{q}}}{\sqrt{\beta_{q}^{\nu_{q}}}\Gamma \left(\nu_{q}\right)}K_{\nu_{q}}\left(\frac{2x_{m}}{\sqrt{\beta_{q}}}\right)\right)}^{-1}\frac{4x^{\nu_{q}}}{\beta_{q}^{\frac{\nu_{q}+1}{2}}\Gamma \left(\nu_{q}\right)}K_{\nu_{q}-1}\left(\frac{2x}{\sqrt{\beta_{q}}}\right),\;0\leq x\leq x_{m}$$

for the K distribution and as

(A6) $$p\left(x=|d|\right)={\left(1-\frac{4^{\nu_{p}}}{{\left(\beta_{p}x_{m}^{2}+4\right)}^{\nu_{p}}}\right)}^{-1}\frac{x\beta_{p}\Gamma \left(\nu_{p}+1\right)}{2{\left(\frac{\beta_{p}}{4}x^{2}+1\right)}^{\nu_{p}+1}\Gamma \left(\nu_{p}\right)},\;0\leq x\leq x_{m}$$

for the GP distribution.

## Appendix B

Samples from truncated K (A5) and GP (A6) distributions with shape and scale parameters $\nu_{p},\nu_{q}$, and $\beta_{p},\beta_{q}$, respectively, as well as with maximum value $x_{m}$, are generated based on the compound nature of these densities. Specifically, speckle x follows normalised complex Gaussian noise and if the background (or texture) ζ follows an inverse gamma distribution (A3), the compound distribution $d=\left|x\right|\sqrt{\zeta }$ follows a GP distribution. If the texture follows a gamma distribution (A1), *d* follows a K distribution. Leveraging this approach, a naive accept–reject method [53] for generating samples from truncated K and GP distributions is outlined in Table A1. The random number generators for gamma and normal distributions, among others, are provided as built-in functions in the Scilab computation engine [54], which is utilised throughout the section on numerical examples.

**Table A1:** Algorithm for generating *n* samples from truncated K and GP distributions.

Step	Description
1	i=1
2	If distribution is GP, then generate sample $\zeta_{i}$ from gamma distribution (A1) with shape and scale parameters $\nu_{p},\beta_{p}$, else, generate sample $\zeta_{i}$ from gamma distribution with shape and scale parameters $\nu_{q},\beta_{q}$
3	If distribution is GP, then $\zeta_{i}=1/\zeta_{i}$, else, continue
4	Generate in-phase sample $x_{I_{i}}$ from zero-mean Gaussian distribution with variance $\zeta_{i}$, i.e., $x_{I_{i}}∼N\left(0,\zeta_{i}\right)$
5	Generate quadrature-phase sample $x_{Q_{i}}$ from zero-mean Gaussian distribution with variance $\zeta_{i}$, i.e., $x_{Q_{i}}∼N\left(0,\zeta_{i}\right)$
6	Amplitude of compound distribution is $d_{i}=\left|x_{I_{i}}+ix_{Q_{i}}\right|$
7	If $d_{i}\leq x_{m}$, then i=i+1, else, go to Step 2
8	If i≤n, go to Step 2, else, stop

This approach, although functional, is relatively inefficient. For both the K and GP distributions, generating 100,000 samples that follow truncated K or GP distributions requires approximately seven times more computational effort compared to the non-truncated case.
